# Prevalence and Characterization of *Staphylococcus aureus* and Methicillin-Resistant *S. aureus* from Different Retail Raw Meats in Shandong, China

**DOI:** 10.3390/microorganisms13061361

**Published:** 2025-06-11

**Authors:** Xiaonan Zhao, Bingyu Hou, Zijing Ju, Wenbo Wang

**Affiliations:** 1 Institute of Animal Science and Veterinary Medicine, Shandong Academy of Agricultural Sciences, Jinan 250100, China; zhaoxiaonan1214@163.com (X.Z.); houbingyu1003@163.com (B.H.); 2 CAS Key Laboratory of Microbial Physiological and Metabolic Engineering, Chinese Academy of Sciences, Beijing 100101, China; 3 Institute of Agricultural Quality Standards and Testing Technology, Shandong Academy of Agricultural Sciences, Jinan 250100, China

**Keywords:** *Staphylococcus aureus*, retail raw meat, antimicrobial resistance, molecular epidemiology

## Abstract

*Staphylococcus aureus* is an important cause of food intoxication, which has the potential to induce diverse infections, toxinoses and life-threatening diseases among humans and animals. This study investigated the prevalence, antimicrobial resistance, and genetic diversity of *S. aureus* and methicillin-resistant *S. aureus* (MRSA) in retail raw meat from Shandong (March 2021–October 2022). The distribution of virulence genes, antimicrobial susceptibility, and genetic diversity of these isolates were analyzed. From a total of 442 samples, 87 (19.7%) *S. aureus* and 11 (2.5%) MRSA were isolated. According to the antimicrobial susceptibility testing, it was found that all the *S. aureus* isolates were resistant to at least one antimicrobial. Most isolates (95.9%) were resistant to penicillin, with high resistance to ampicillin (82.7%) and multidrug resistance in 76.5% of cases. One isolate could simultaneously resist eleven antimicrobials (ERY-CLI-GEN-SMZ-FFC-PEN-PRL-AMC-CIP-TET-AMP). In contrast, all the isolates showed sensitivity to vancomycin. The most prevalent virulence gene was *sed*, accounting for 10.2%, followed by *sec* (8.2%). Regarding genetic polymorphism, these isolates were divided into 21 different sequence types (STs) using multilocus sequence typing (MLST) and 33 staphylococcal protein A (*spa*) types using spaTyper 1.0 tool. The most prevalent sequence types were ST398 (22.4%), followed by ST7 (20.4%), while ST59, ST1, ST188, ST9, ST398, and ST7 were observed in MRSA isolates. The most prevalent *spa* types were t034 (15.3%), followed by t899 (10.2%), while t441, t127, t184, t899, t034, and t091 were observed in MRSA isolates. In conclusion, our study highlights the high prevalence of *S. aureus* and MRSA in different retail raw meats in Shandong. This poses a potential threat to food safety and underscores the need for enhanced surveillance and stricter antibiotic control measures.

## 1. Introduction

*Staphylococcus aureus* is a facultatively anaerobic, Gram-positive, non-motile, non-spore-forming, round-shaped bacterium that has been found in previous research to exhibit high salt and sugar tolerance, as well as the ability to grow at water activity levels as low as 0.83. Staphylococcal infections do not develop on healthy skin. However, if allowed to enter the bloodstream or internal tissues, *S. aureus* can cause a variety of potentially serious infections, such as osteomyelitis, sepsis, and toxic shock syndrome [1]. The contamination of food with *S. aureus* can begin on farms through infection or colonization of livestock and farm workers. It then spreads through the food chain due to inadequate human handling of food products [2,3]. Staphylococcal foodborne poisoning is a common foodborne disease worldwide. In food samples, the majority of contamination has been detected in raw meat, posing a risk to consumers and causing significant economic losses to producers. In addition, chicken has been reported to be much more frequently contaminated with *S. aureus* as compared to red meat [4,5]. It is postulated that 20–25% of foodborne bacterial epidemics in China results from *S. aureus* [6]. In the U.S., *S. aureus* leads to 241,000 cases of illness each year, resulting in a large economic load [7].

*S. aureus* exhibits the ability to produce a variety of extracellular proteins, referred to as exotoxins, including enterotoxins, hemolysins, and leukocidins, that can mediate hemolytic and cytotoxic activities, which impede phagocytosis. The particular noteworthy exotoxins in this bacterium are the Staphylococcal enterotoxins (SEs), which are the primary cause of food poisoning outbreaks [8]. The major SEs (*sea*, *seb*, *sec*, *sed*, and *see*) are responsible for approximately 95% of *S. aureus*-related outbreaks. Even after bacterial inactivation treatments, these toxins remain stable and resistant to human digestive enzymes [9]. As superantigens, they play a role in activating a great number of T cells, causing proinflammatory cytokine release [10]. Therefore, even a small amount of SEs (~20–100 ng) can lead to staphylococcal food poisoning in humans [11].

The excessive use of antimicrobials in farm animals has contributed to the emergence of antimicrobial-resistant *S. aureus* in food samples [12]. Drug-resistant *S. aureus* has been linked to multiple food poisoning outbreaks and is recognized as a global health threat due to its impact on treatment efficacy. At least 25% of the foodborne isolates show resistance to at least one class of antimicrobials. An estimate of current annual deaths due to antimicrobial resistance is 700,000 and, by the year 2050, deaths by complications due to infectious diseases will exceed 10 million annually [13]. MRSA is a significant global public health concern due to its high resistance to penicillin, frequent multidrug resistance, and association with increased mortality rates [14]. It has been designated as one of the high-priority antibiotic-resistant pathogens by the World Health Organization. MRSA resistance is mediated by the staphylococcal cassette chromosome *mec* (SCCmec), which encodes a modified penicillin-binding protein (PBP2a). Multiple studies have clearly documented foodborne MRSA outbreaks worldwide [15,16]. Therefore, stricter regulations on antimicrobial use in food-producing animals are essential to curb the development of antimicrobial resistance.

Conventional treatments, such as antimicrobial agents, heat or chemical preservatives (nitrite, phosphate, and organic acids), have been widely used to prevent *S. aureus*-related outbreaks in food products. However, they usually require high energy consumption, contribute to antimicrobial resistance, and deteriorate the nutritional and sensory quality of food. Of greater concern, some of them may have negative impacts on human health [17]. Therefore, it is urgent to explore the prevention and control strategies of *S. aureus,* such as bacteriophage therapy, which should be put on the agenda.

In recent years, China has gradually restricted antimicrobial use in food-producing animals [18]. However, the impact of these policies on retail raw meat remains unclear. Limited studies have examined *S. aureus* and MRSA contamination in retail raw meat in Shandong. Thus, this study aimed to identify *S. aureus* and MRSA in retail raw meat in Shandong from March 2021 to October 2022 and characterize their antimicrobial resistance, virulence genes, and genetic diversity. These findings provide essential data for the prevention and control of *S. aureus* and MRSA contamination in retail meat.

## 2. Materials and Methods

### 2.1. Sample Collection

**Figure 1:**
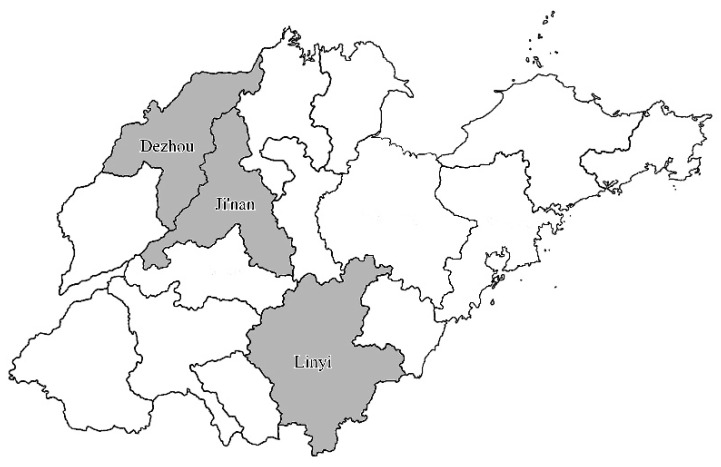
Distribution of *S. aureus*-positive samples in retail raw meat collected from three main regions.

From March 2021 to October 2022, a total of 442 samples of retail raw meat were obtained from eleven large supermarkets (sized around 5000 m^2^) located in Linyi, Jinan, and Dezhou regions of Shandong Province (Figure 1). The samples included pork (*n* = 158), chicken (*n* = 110), beef (*n* = 55), mutton (*n* = 54), and duck meat (*n* = 65) (Table 1). The sample collection conformed to the cluster random sampling principle. The retail raw meat samples were collected aseptically using sterile forceps and placed in sterile sample bags. The samples were maintained at a temperature of 4 °C during transport. Microbiological analyses were performed immediately upon arrival at the laboratory.

### 2.2. Isolation, Identification, and Enumeration of S. aureus and MRSA

The enrichment and isolation of *S. aureus* were carried out according to the method described previously, with some modifications [19]. Briefly, 25 g of samples was homogenized and mixed with 225 mL of sterile-buffered peptone water (BPW, Beijing Land Bridge Technology Ltd., Beijing, China). A single loopful of enriched culture was then plated onto Baird-parker agar plate (Hope Bio-Technology Co., Ltd., Qingdao, China). Subsequently, the plate was cultured under aerobic conditions at a temperature of 37 °C for 18–24 h. The suspected colonies were further plated on blood agar, and upon overnight incubation, they were identified using matrix-assisted laser desorption/ionization time-of-flight mass spectrometry (Bruker Daltonik GmbH, Bremen, Germany). All the isolates were stored at −80 °C in 30% (*v*/*v*) aqueous glycerol until further use. To confirm MRSA, all *S. aureus* isolates were examined for the carriage of *mecA* gene using PCR method as previously described [19]. The amplicons were detected at 90 V for 120 min on a 1% agarose gel and then visualized under UV-transilluminator gel imaging system.

### 2.3. Antimicrobial Susceptibility Testing

The agar dilution method was utilized to test the antimicrobial susceptibility of all *S. aureus* on Mueller-Hinton agar (Hopebiol, Qingdao, China) plates. Eighteen antimicrobials from ten antimicrobial classes were chosen based on previous studies [6,19], with all reagents procured from Thermo Fisher Scientific (Shanghai, China). The antimicrobials used were penicillin (PEN, 0.06–32 μg/mL), cefotaxime (CF, 0.25–64 μg/mL), ceftiofur (CEF, 0.25–8 μg/mL), oxacillin (OXA, 0.12–8 μg/mL), ampicillin (AMP, 0.12–64 μg/mL), clindamycin (CLI, 0.06–4 μg/mL), pirlimycin (PRL, 0.12–4 μg/mL), ciprofloxacin (CIP, 0.12–4 μg/mL), sulfamethoxazole (SMZ, 0.125–32 μg/mL), trimethoprim/sulfamethoxazole (SXT, 0.12/2.38–4/76 μg/mL), vancomycin (VAN, 0.5–32 μg/mL), doxycycline (DOX, 0.12–8 μg/mL), erythromycin (ERY, 0.25–8 μg/mL), tetracycline (TET, 0.25–16 μg/mL), florfenicol (FFC, 2–32 μg/mL), amoxicillin/clavulanic acid (AMC, 0.12/0.06–16/8 μg/mL), rifampicin (RFP, 0.12–4 μg/mL), gentamicin (GEN, 0.5–16 μg/mL). In brief, resurgent strains were cultured for about 40 h. Then, the bacterial concentration was adjusted to OD600 0.1–0.3. Subsequently, 100 μL aliquots of standardized suspensions were spread onto Mueller-Hinton (MH) agar plates containing serial two-fold dilutions of antimicrobial agents. Plates were incubated aerobically at 37 °C for 18–24 h. The minimum inhibitory concentrations (MICs) were defined as the lowest antimicrobial concentration that inhibited visible bacterial growth. *Escherichia coli* ATCC 25922 and *S. aureus* ATCC 29213 were used as quality control strains. MDR was defined as resistance to at least one agent in three or more antimicrobial categories [19]. The results were interpreted according to Clinical and Laboratory Standards Institute (CLSI) documents VET01-S and M100 [20].

### 2.4. Detection of Staphylococcal Enterotoxin Genes

Bacterial DNA was extracted using TIANamp bacterial DNA extraction kits (TianGen DNA Kit, Beijing, China) following the manufacturer’s instructions. Overnight *S. aureus* cultures in Brain Heart Infusion Broth (BHI) were centrifuged at 10,000 rpm for 1 min in labelled 2 mL safe-lock tubes. The pellets were resuspended in 180 μL of digestion buffer (20 mM Tris, pH 8.0; 2 mM disodium ethylenedi-aminetetraacetate (Na_2_-EDTA); 1.2% Triton; 20 mg/mL lysozyme) obtained from Tiangen Biotech Co., Ltd. (Beijing, China), and incubated at 37 °C for 30 min. Then, 20 μL of proteinase K and 220 μL of genomic lysis/binding buffer were added, and the mixture was vortexed for 15 s. The digestion mixtures were incubated at 55 °C for 30 min, and then 220 μL of absolute ethanol was added to each tube and mixed well. The obtained solution and precipitate from each digestion were added to individual adsorption column and centrifuged at 12,000 rpm for 30 s to remove the waste. Then, each column was washed twice more by adding 500 μL of protein removal buffer and centrifuging at 12,000 rpm for 30 s to remove the waste, and each rinse solution was evaporated by drying at 50 °C. The DNA was eluted from the adsorption column by adding 100 μL of Tris-EDTA (TE) elution buffer followed by centrifugation at 12,000 rpm for 30 s. After quantifying and assessing the purity of the eluted DNA using a NanoDrop 2000c spectrophotometer (Thermo Fisher Scientifc Inc., Waltham, MA, USA), the DNA was preserved at −20 °C for subsequent analysis.

As previously described, PCR analysis was performed on all *S. aureus* isolates to detect the presence of five classical enterotoxin genes (*sea*, *seb*, *sec*, *sed*, and *see*) [21]. The PCR reaction mixture (25 μL per reaction) consists of 12.5 μL of 2 × MIX (Thermo Fischer Scientific, Waltham, MA, USA), 10 pmol of each primer, and 100 ng template DNA. The PCR amplification conditions were as follows: an initial DNA denaturation at 94 °C for 4 min, 32 cycles at 94 °C for 60 s, 55 °C for 30 s and 72 °C for 90 s, and a final extension of 5 min at 72 °C. The amplicons were detected at 90 V for 120 min on a 1% agarose gel and then visualized under UV-transilluminator gel imaging system.

### 2.5. MLST and Spa Typing

All *S. aureus* isolates were characterized by the MLST and *spa* typing. MLST analysis was conducted based on seven loci to characterize strains by assigning them unique sequence types. The seven housekeeping loci that specifically target *S. aureus* comprise carbamate kinase (*arc*), shikimate dehydrogenase (*aro*), glycerol kinase (*glp*), guanylate kinase (*gmk*), phosphate acetyltransferase (*pta*), triosephosphate isomerase (*tpi*) and acetyl coenzyme A acetyltransferase (*yqi*) [22]. PCR amplification of the seven house-keeping genes of each *S. aureus* isolate was performed according to the protocols on the MLST database (https://pubmlst.org/, accessed on 13 September 2021). The fragments of amplified genes were then sequenced by ThermoFisher Scientific Corporation (Shanghai, China). Through the *S. aureus* MLST database, the allelic profiles and STs of each isolate were identified. To analyze the distribution of STs, a minimum spanning tree was constructed using BioNumerics software, version 6.5 (Applied Maths, Kortrijk, Belgium). For *spa* typing, the polymorphic X region of *spa* was amplified and sequenced using a specific primer set [23]. The sequences were submitted to the Ridom Spa Server (http://spa.ridom.de/, accessed on 25 February 2022) to determine the *spa* types based on the number and arrangement of tandem repeat sequences. The genetic relationships among all isolates were visualized as a phylogenetic tree based on *spa* types using the MEGA 7.0 software.

### 2.6. Statistical Analyses

All statistical analyses were performed using SPSS 15.0 (SPSS Inc., Chicago, IL, USA). The chi-square test was used to compare the prevalence of *S. aureus* and MRSA isolated from duck, pork, beef, mutton, and chicken, with *p* < 0.05 considered statistically significant. Heterogeneity in resistance rates was evaluated using Cochran’s Q test (threshold: *p* < 0.05). Significant outcomes were subjected to Marascuilo post hoc testing with multiplicity-adjusted α = 0.05, with results summarized by letter-based significance grouping (shared letters indicate non-significance).

## 3. Results

### 3.1. Prevalence of S. aureus and MRSA

Among 442 samples, 87 (19.7%) were positive for *S. aureus*. Additionally, 11 (2.5%) samples were *mecA*-positive and identified as MRSA. Among five kinds of retail raw meats, the contamination rate of *S. aureus* was highest in duck meat (26.2%), followed by pork (23.4%), beef (18.2%), mutton (14.8%), and chicken meats (13.6%), while pork had the highest contamination rate of MRSA at 3.8%, followed by mutton (1.9%), beef (1.8%), chicken (1.8%), and duck (1.5%). No statistically significant differences were found in the isolation rates of *S. aureus* and MRSA among duck, pork, beef, mutton, and chicken meats (*p* > 0.05). Detailed information on all isolates is presented in Table 1 and Table S1.

### 3.2. Antimicrobial Susceptibility Testing

**Figure 2:**
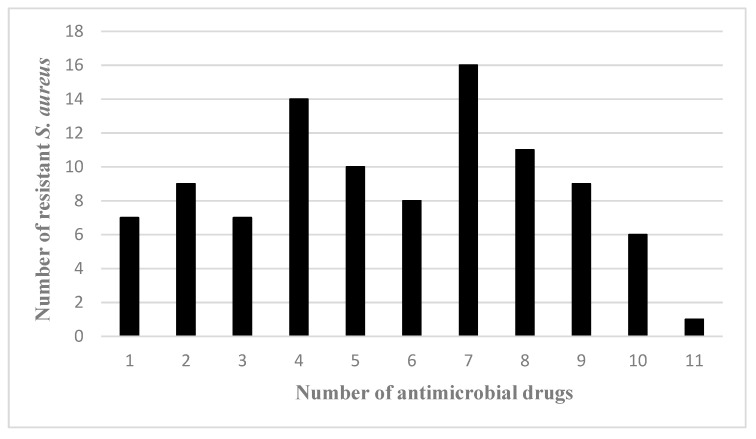
Frequency distribution of antimicrobial agent resistance patterns of *S. aureus*. The x-axis shows the number of antimicrobial drugs to which the isolates were resistant, and the y-axis shows the number of resistant *S. aureus* isolates.

The susceptibility of 98 *S. aureus* isolates to 18 antimicrobials was tested. Among the tested antimicrobials, all of the *S. aureus* isolates were susceptible to vancomycin. Resistance to penicillin was the most common (95.9%), followed by ampicillin (82.7%), erythromycin (62.2%), clindamycin (53.1%), and tetracycline (52.0%) (Table 2). According to the statistical analysis, penicillin and ampicillin exhibited significantly higher resistance rates than all other tested antimicrobial agents (all group comparisons *p* < 0.001). Conversely, vancomycin, cefotaxime, doxycycline, and rifampicin demonstrated the lowest resistance rates. Each of the *S. aureus* isolates exhibited resistance to at least one antimicrobial agent. Furthermore, 76.5% of isolates were MDR. Notably, one (1.0%) isolate could simultaneously resist eleven antimicrobials (ERY-CLI-GEN-SMZ-FFC-PEN-PRL-AMC-CIP-TET-AMP), six (6.1%) isolates could simultaneously resist ten antimicrobials, nine (9.2%) isolates could simultaneously resist nine antimicrobials, and eleven (11.2%) isolates could simultaneously resist eight antimicrobials (Figure 2 and Table S1).

### 3.3. Virulence Gene Distribution of S. aureus

From the 98 analyzed *S. aureus* isolates, five classical enterotoxin genes (*sea*, *seb*, *sec*, *sed*, and *see*) were identified. The results indicated that the most prevalent virulence gene was *sed*, with a prevalence of 10.2%, followed by *sec* (8.2%), *seb* (7.1%), and *sea* (5.1%). Notably, none of the isolates harbored the *see* gene, as presented in Table 3.

### 3.4. MLST and Spa Typing

**Figure 3:**
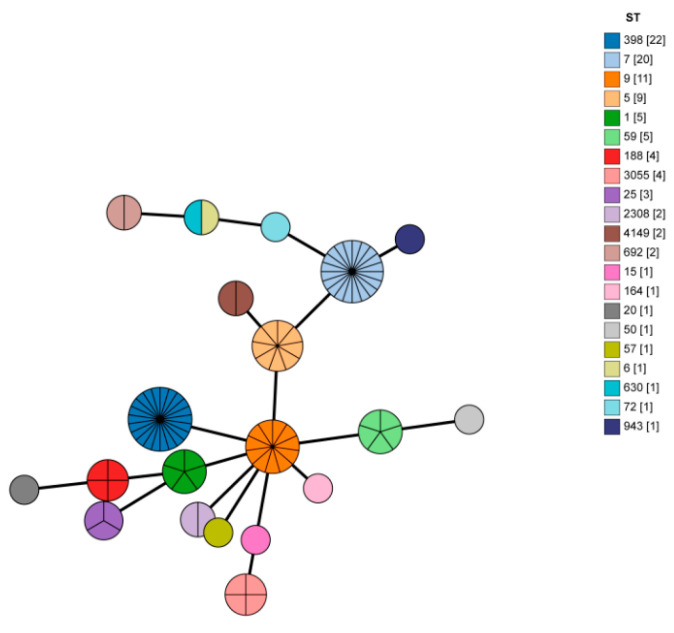
Minimum spanning tree of *S. aureus* isolates from different retail raw meat in Shandong. The minimum spanning tree was constructed by Bionumerics using MLST data. Each circle represents a different ST and the circle size indicates the number of strains. The numbers within [ ] indicate the number of bacterial strains.

**Figure 4:**
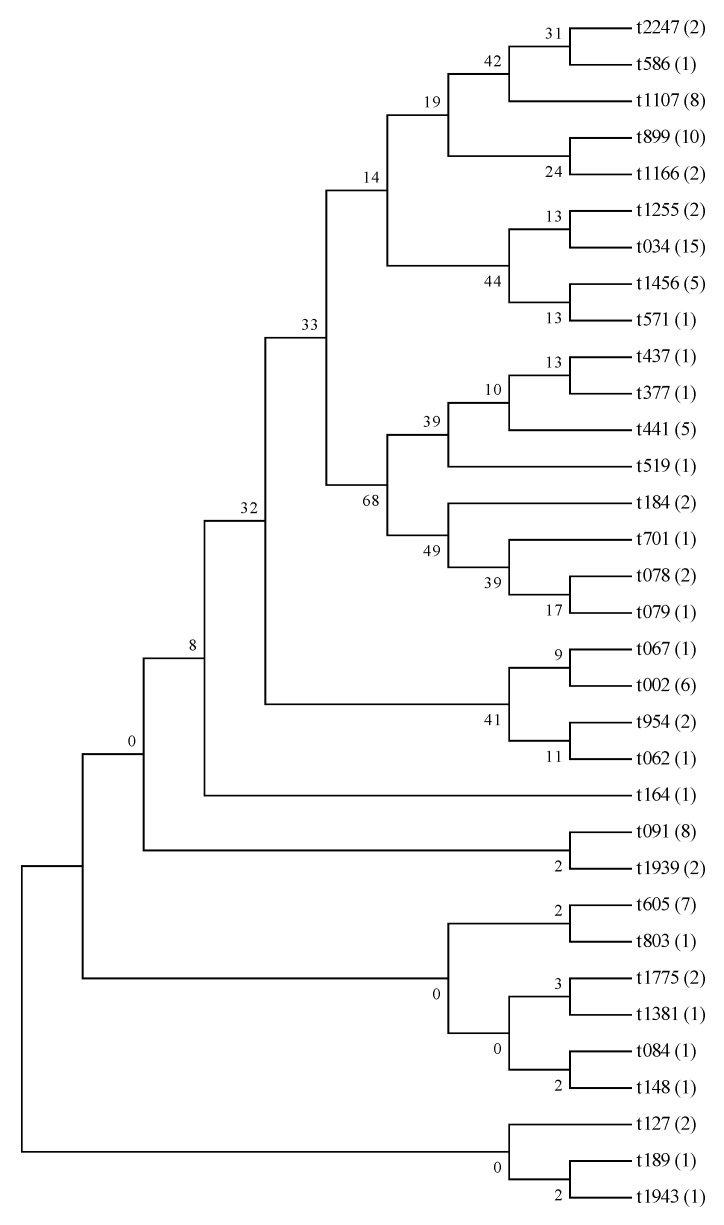
Phylogenic tree of the 98 isolates based on *spa* typing. A total of 98 *S. aureus* isolates with 33 unique *spa* types were clustered using the software MEGA 6.0.

Among the 98 isolates that were analyzed using the MLST (https://pubmlst.org/, accessed on 13 September 2021), 21 different STs were identified, as shown in Figure 3 and Table S1. ST diversity was exhibited in all types of meat. The most prevalent sequence types were ST398 (22.4%), followed by ST7 (20.4%), ST9 (11.2%), and ST5 (9.2%). Furthermore, among the MRSA isolates, the STs were determined to be ST59, ST1, ST188, ST9, ST398, and ST7. A total of 33 *spa* types were obtained with the application of the spaTyper 1.0 tool, as depicted in Figure 4 and Table S1. Among the *spa* types, t034 was the most common, with a prevalence rate of 15.3%, followed by t899 (10.2%), t1107 and t091 (8.2%). Furthermore, the *spa* types identified among the MRSA isolates were t441, t127, t184, t899, t034, and t091.

## 4. Discussion

*S. aureus* is regarded as a bacterium that draws significant attention in the animal–food–human chain because it has the ability to develop resistance to antimicrobial agents and can produce various heat-stable enterotoxins. The present study demonstrated that *S. aureus* could be found in different retail raw meats, with a prevalence rate of 19.7%, which was consistent with the previously reported prevalence of 21.2% [11] but lower than those of 35.0% [24] and 39.2% [25]. This study revealed that the prevalence rate of *S. aureus* was lower in chicken, while it was higher in duck meat. This may be due to the limitation of the sampling quantity.

Dubbed a “superbug”, MRSA is resistant not only to methicillin (methicillin and oxacillin) but also to other categories of antimicrobials, such as macrolides, chloramphenicol, aminoglycosides, tetracyclines, and lincosamides, so it causes a threat to public health because it is difficult to treat, making patients’ treatment options limited and making the search for new compounds active against it inevitable [26]. The detection of MRSA strains in Chinese food products has been reported previously for a variety of foods, such as raw meat, rice flour, vegetable salads, sandwiches, meat products, and eggs [6,10,24]. This study demonstrated that the prevalence of MRSA was 2.5%. It was lower than the prevalence rates previously reported by Titouche et al. (2020) [27], with 8.3% for MRSA in food products, and by Seow et al. (2021) [28], which was 8.0% among cooked food. Conversely, the MRSA contamination rate was higher than previously reported from retail foods (0.67%) and bulk tank milk (0.7%) [19,29]. The discrepant isolation rates of *S. aureus* and MRSA across various studies might be ascribed to factors such as geographical origin, environmental conditions, as well as the isolation and identification methods. The results indicated that both *S. aureus* and MRSA were prevalent in different retail raw meats in Shandong. The occurrence of MRSA in these meats has raised significant concerns regarding food and its possible role as a reservoir for MRSA.

Over the past few decades, *S. aureus,* with resistance to a variety of antimicrobials, has emerged. Penicillin and its derivatives proved to be extremely effective when first utilized for treating staphylococcal infections. Nevertheless, with the overuse of antimicrobials, penicillin-resistant *S. aureus* strains quickly emerged and rapidly spread globally [30]. Today, multiple antimicrobial-resistant strains of *S. aureus* are spreading rapidly around the world, which raises serious health concerns. In this study, all *S. aureus* isolates demonstrated resistance to at least one antimicrobial agent. Notably, a high level of resistance was shown towards penicillin, tetracycline, ampicillin, and erythromycin, consistent with previous reports [10,31,32]. As an antibiotic that is frequently utilized, penicillin is widely adopted for controlling and treating bacterial infections in farm settings. Thus, high resistance to penicillin can be observed in *S. aureus* isolates. Moreover, 76.5% of isolates were MDR, with all MRSA isolates showing MDR. This was similar to the report that 87.2% of *S. aureus* isolates obtained from retail raw chicken were MDR [10]; however, this is higher than that previously reported for ready-to-eat foods by Zhou et al. (2024) [33]. The prevalence of MDR documented in other countries was as follows: 96.8% in Turkey [11], 82% in South Africa [34], and 87.5% in Ghana [35]. The variation in resistance rates may be ascribed to the antibiotic usage among humans and animals within a specific area. These results indicated that in order to treat antimicrobial-resistant *S. aureus*, we should actively develop new antimicrobials and flexibly combine existing ones to treat infections and delay the development of *S. aureus* resistance to available antimicrobials.

Detecting *S. aureus* that carries genes responsible for encoding SEs serves as a significant indicator, highlighting the risks entailed by food contamination [36]. In this study, the *sed* gene was the most prevalent. It was similar to other reports that *sed* was the SE-encoding gene with the highest occurrence in *S. aureus* isolates within foods and raw milk [19,37]. In contrast, it has been reported that *sea* was the predominant SE in many countries, accounting for about 80% [38,39]. In the current study, the prevalence of *seb* was 7.1%, which has been documented to be associated with staphylococcal foodborne poisoning and bovine mastitis [40]. Similar to previous findings, no isolate was found to harbor the *see* gene [19,25,41]. The presence of diverse SEs in *S. aureus* and MRSA obtained from different retail raw meats is an important indicator that highlights the risk associated with food contamination.

In the current study, molecular epidemiology of the *S. aureus* isolates collected from different retail raw meats in Shandong was assessed by MLST and *spa* typing to investigate the evolutionary relationship. According to previous reports, STs were commonly correlated with food-related disease outbreaks, thereby highlighting their significance in public health surveillance [42]. Among the STs identified in this study, ST398 was the most prevalent sequence type, consistent with previous findings [43]. In contrast, Ou et al. (2020) reported that ST7 was the predominant sequence type within animal-based food [4]. Moreover, a prior study showed that between 2016 and 2018, the occurrence frequency of MRSA ST398 found in human bloodstream infections was 1.3% [44]. Notably, in our study, two MRSA ST398 isolates were obtained, which were likely to be directly transmitted to humans via the food chain [45,46]. Previous research also indicated that ST5 had a greater ability in terms of hemolysis and adhesion, leading to more critical infections in the murine abscess model [47]. In line with a prior report, ST5 was distributed in different retail raw meats in our study as well [48]. Overall, these findings regarding different STs further emphasize the significance of the continuous monitoring of *S. aureus* in food sources to safeguard public health.

Several molecular typing methods were used to characterize the isolates, including MLST and *spa* typing. MLST is a DNA sequencing technology that uses sequence analyses of housekeeping genes to discriminate between isolates. MLST also offers the advantage that it is highly reproducible, which makes it an excellent tool for global comparisons of population structures [22]. *spa* typing is specific to staphylococci and analyzes the polymorphisms in the protein A gene [23]. A previous study showed associations between diverse STs and *spa* types [49]. In the present study, t034 was identified as the most prevalent *spa* type, similar to a previous study [50]. In addition, among the *S. aureus* isolates with t034, the majority belonged to ST398, which coincides with a previous study [43]. It is worth noting that MRSA ST398-t034, which is usually linked to swine and farmers in Europe, was found in beef and pork samples within this study [51]. However, according to a previous report [52], the dominant *spa* types for isolates from food products were t002, t091, t127 and t189, which was different from the results of our study. The prevalence of genotypes and the clonal spread among *S. aureus* might be determined by geographical location.

## 5. Conclusions

This study analyzed the prevalence, antimicrobial resistance profiles, and genetic diversity of *S. aureus* (19.7%, *n* = 87) and MRSA (2.5%, *n* = 11) in retail raw meat samples in Shandong Province, China. Antimicrobial resistance testing revealed high resistance rates to penicillin, ampicillin, erythromycin, clindamycin, and tetracycline among isolates. Notably, the livestock-associated MRSA strain ST398-t034 was identified. MDR posed a critical issue among the isolates, and the occurrence rate of enterotoxin genes was considerable. Hence, monitoring the usage of antimicrobial agents in farm animals becomes crucial. Effective reductions in staphylococcal contamination levels could be achieved by improving sanitation and hygiene procedures. Our research not only underlines the requirement for sustained surveillance of retail raw meat but also provides important insights by providing corresponding data and scientific proof. Such contributions will facilitate the surveillance of the dissemination of *S. aureus* and MRSA and contribute to the development of efficient strategies for ensuring food safety. Furthermore, regular screening of animals, farmers, farm and slaughterhouse environments, and thorough cooking of meat, should be implemented to detect the emergence and persistence of pathogenic *S. aureus* strains, to prevent dissemination to humans.

## Figures and Tables

**Table 1:** Isolation frequency of *S. aureus* and MRSA from different retail raw meats.

Sample Type	No of Samples	No *S. aureus* (%)	No MRSA (%)
Pork	158	37 (23.4)	6 (3.8)
Chicken	110	15 (13.6)	2 (1.8)
Beef	55	10 (18.2)	1 (1.8)
Mutton	54	8 (14.8)	1 (1.9)
Duck	65	17 (26.2)	1 (1.5)
Total	442	87 (19.7)	11 (2.5)

**Table 2:** Number and percentage of antimicrobial resistance of *S. aureus* (87 *S. aureus* + 11 MRSA) isolated from different retail raw meats.

Antimicrobial Class	Antimicrobial Agent	No. of *S. aureus* (%)	Significance Group ^1^
β-Lactams	Penicillin	94 (95.9)	a
	Amoxicillin/clavulanic acid	17 (17.3)	b
	Ceftiofur	9 (9.2)	jkl
	Cefotaxime	2 (2.0)	klm
	Oxacillin	10 (10.2)	ijk
	Ampicillin	81 (82.7)	b
Macrolides	Erythromycin	61 (62.2)	cd
Lincomycin	Clindamycin	52 (53.1)	cde
	Pirlimycin	45 (45.9)	efg
Quinolones	Ciprofloxacin	25 (25.5)	fgh
Sulfonamides	Sulfamethoxazole	47 (48.0)	def
	Trimethoprim/sulfamethoxazole	11 (11.2)	jkl
Glycopeptide	Vancomycin	0	m
Tetracyclines	Doxycycline	3 (3.1)	lm
	Tetracycline	51 (52.0)	c
Chloramphenicol	Florfenicol	11 (11.2)	jkl
Rifampicin	Rifampicin	3 (3.1)	lm
Aminoglycosides	Gentamicin	30 (30.6)	fgh

^1^ Groups denoted by the same letter indicate no significant difference (Marascuilo procedure, α = 0.05).

**Table 3:** Distribution of genes encoding enterotoxins in *S. aureus* isolated from different retail raw meats.

Genes	Pork (*n* = 43)	Chicken (*n* = 17)	Beef (*n* = 11)	Mutton (*n* = 9)	Duck (*n* = 18)	Total (*n* = 98)	Percentage (%)
*sea*	4	1	0	0	0	5	5.1
*seb*	4	2	0	1	0	7	7.1
*sec*	4	2	0	1	1	8	8.2
*sed*	3	4	0	1	2	10	10.2
*see*	0	0	0	0	0	0	0

## Data Availability

The original contributions presented in this study are included in the article/Supplementary Materials. Further inquiries can be directed to the corresponding authors.

## Supplementary Materials

The following supporting information can be downloaded at: https://www.mdpi.com/article/10.3390/microorganisms13061361/s1, Table S1: Resistance phenotype, ST, and *spa* in *S. aureus* from Different Retail Raw Meats.
